# Parkinson's disease and routine traffic stops: An exploration of patient experiences and perceptions

**DOI:** 10.1177/1877718X251356514

**Published:** 2025-07-15

**Authors:** Rebecca Eilish Irvine, Alexis P Kruger, Alfred Quaicoe, Roshni Kotwani, Anna DePold Hohler, Okeanis Vaou

**Affiliations:** 1 School of Medicine, Imperial College London, London, UK; 2 Chobanian & Avedisian School of Medicine, Boston University, Boston, MA, USA; 3 School of Medicine, Tufts University, Boston, MA, USA; 4 Dr. Kiran C. Patel College of Osteopathic Medicine, NSU Clearwater, FL, USA; 5 Department of Neurology, St. Elizabeth Medical Center, Boston, MA, USA; 6 Department of Neurology, University of Texas Health Sciences San Antonio, San Antonio, TX, USA

**Keywords:** motor system, movement analysis, quality of life

## Abstract

Parkinson's disease (PD) is a chronic neurodegenerative disorder which can impair patients’ ability to complete complex motor skills, such as driving. There is currently a lack of research into the effectiveness of police officers discerning PD symptoms from those exhibited by alcohol intoxication during traffic stops. We conducted a survey on 58 PD patients and 52 age-matched controls to investigate experiences with police interactions during traffic stops. PD patients had statistically significant (*p* = 0.03204) higher perceptions of mistreatment and misclassification of intoxication. We propose the need for additional training and support on PD for law enforcement officers to ensure fair evaluation.

## Plain language summary

Research has shown that as disease progresses, patients with Parkinson's Disease (PD) have a reduced ability to complete tasks that involve coordination of movement, such as driving. However, it is unknown whether police officers are able to distinguish symptoms of PD from similar driving patterns shown in people who have consumed alcohol prior to driving. In this study, we surveyed 58 people with PD and 52 people of similar age without PD, to investigate their experiences with police officers during routine traffic stops. We found an increase in the perception of mistreatment amongst PD patients, compared to people without PD. Because of this, we propose the importance of more extensive training programs for police officers regarding neurological disorders such as PD, to ensure patients are evaluated fairly and handled appropriately.

Parkinson's disease (PD) is a neurodegenerative disorder affecting millions of people worldwide. PD is characterized by progressive deterioration of motor symptoms, including bradykinesia, resting tremor, and postural instability, significantly impacting quality of life.^1^ A significant concern for PD patients is their ability to drive safely, given the disease's effects on the ability to undertake complex motor tasks.^2^

Traditionally, PD research has focused on the patient's driving capabilities in relation to disease progression.^3,4^ A study comparing the driving abilities of 20 PD patients to 20 age- and sex-matched controls found that PD patients had significantly poorer performances in driving tests.^3^ Additionally, a survey involving 6620 PD patients demonstrated that 15% had reported being in an accident in the past 5 years, with 11% responsible for the collision. The risk of causing accidents was significantly correlated with patients who felt moderately impaired by their PD status.^4^

However, there is a lack of research investigating patients’ real-world experiences of police officers evaluating their driving. Anecdotal evidence demonstrates that some patients believe they have had their PD symptoms mislabeled as being under the influence of toxic substances, specifically alcohol. Adverse police interactions can escalate into unwarranted investigations, incurring unnecessary legal costs and causing emotional distress.^5^ The objective of our study was to investigate the perception of misclassification and mistreatment of PD patients during police engagements at routine traffic stops. We hypothesise that PD patients will experience greater discrimination compared to controls.

58 PD participants were selected based on having a primary diagnosis of PD^1^ and driving history within the last 10 years, recruited consecutively during movement clinic visits. The age range for the PD cohort was primarily 50–90 years old, with 3 participants older than 90 years old. The comparison cohort was comprised of 52 age-matched controls with no self-reported cognitive or physical difficulties, recruited via convenience sampling. Demographic information for each cohort is listed in Table 1.

**Table 1.** Table showing participant demographics and survey responses from PD patients and controls.

	Parkinson's Disease	Controls
Total Participants (n)	58	52
Gender (Male-Female) (n)	43–15	32–20
Race (White-Ethnic minorities) (n)	53–5	49–3
Mean age (y)	69	63
Mean disease duration (y)	7.9	N/A
Reported difficulty driving (n)	19	6
Experienced a traffic stop while driving (n)	26	29
Reported being mistreated or misclassified as intoxicated by law enforcement officers (n)	9	1

Ethical approval was obtained from the Institutional Review Board at St Elizabeth's Medical Center. Informed consent was obtained prior to participation. Questionaries were created by the research team and conducted face-to-face.

The primary objective was the self-reported response to questions about the frequency of police stops and incidents of mishandling during these interactions. Secondary study objective questions collected information on frequency of alcohol use, driving status, subjective difficulty when driving, and history of traffic stops by the police. For PD patients, additional survey questions included motor and non-motor symptom severity and whether disease progression influenced their decision to drive.

Statistical comparisons were completed in ‘R’ version 4.3.2, using Pearson's chi-squared test. Data was normally distributed, and a *p*-valve of 0.05 was used. Data is presented as absolute values in a table format.

The PD cohort had a significantly higher proportion of participants (15.5%) who perceived themselves as being mistreated or misclassified as intoxicated, in comparison to just 1.9% in the control cohort. This difference was statistically significant (*p* = 0.03204). Moreover, 32.8% of PD patients reported difficulty driving compared to 11.5% of controls. However, 44.8% of PD patients reported being pulled over by police at any time, compared to 56.8% of controls (Table 1).

Only one PD patient (1.7%) reported being questioned by police officers about PD. Additionally, three PD patients (5.2%) stated that the adverse police interactions they experienced reduced their driving behavior.

Overall, this study aimed to investigate the perception of mistreatment by police officers during routine traffic stops. We found that PD patients reported a higher perception of misclassification as being intoxicated compared to controls.

The insidious progression of PD, combined with the underlying pathophysiology of neuronal dysfunction of motor pathways, means that difficulties with complex motor activities such as driving can be expected amongst PD patients.^6,7^ While it remains true that the motor effects of PD can impair patients’ driving capabilities, we highlight that PD patients report an increased rate of being erroneously viewed and treated as having driven under the influence of alcohol or other toxic substances.

These findings highlight significant gaps in the management of patients with neurodegenerative disorders by law enforcement officers. Escalation of police interactions has the potential to impact mental health^5^ and may lead to increased social stigma and anxiety, and decreased confidence in mobility and independence. This is evident in our study, with three PD patients indicating that adverse interactions with police officers resulted in decreased driving behaviors. This is of particular importance given that PD patients experience higher-than-normal rates of anxiety disorders.^8^

Currently, police training curriculums include limited information on how to approach and treat patients suffering from neurodegenerative diseases, namely Alzheimer's and Parkinson's Disease.^9^ The results of this study suggest that law enforcement officers may benefit from additional training and support. For example, encouragement to inquire about neurodegenerative disorders, coupled with a digital support system to quickly see whether the condition may impact movement or communication. Future research could be conducted to investigate the views and perceptions of law enforcement officers.

Moreover, further research with larger sample sizes should be conducted to examine objective measures of traffic stops and police interactions with PD patients compared to the general public, as well as investigate PD patients’ safety in driving throughout the disease course. Additionally, both PD patients and controls are susceptible to recall bias in answering survey questions. In particular, PD patients are at increased risk of having impaired cognition, including memory, as part of the disease progress.^10^ However, in this study, cognitive testing was not conducted.

Nonetheless, while we acknowledge the challenges of practical implementation, this study raises awareness of the potential mishandling by police officers and indicates a need to evaluate current training programs. By educating law enforcement officers on the symptoms associated with neurodegenerative disease and its effects on driving, we can ensure PD patients are evaluated appropriately and fairly.

To conclude, our findings provide preliminary evidence that PD patients may experience an increased perception of mistreatment, discrimination and bias by law enforcement officers. Additional research is crucial to raise awareness of the potential for unjustified police investigations and charges from routine traffic stops.

## References

[1] Kalia LV Lang AE . Parkinson's disease. Lancet 2015; 386: 896–912. 25904081 10.1016/S0140-6736(14)61393-3

[2] Uc EY Rizzo M O’Shea AMJ , et al. Longitudinal decline of driving safety in Parkinson disease. Neurology 2017; 89: 1951–1958. 29021353 10.1212/WNL.0000000000004629 PMC5679414

[3] Heikkilä VM Turkka J Korpelainen J , et al. Decreased driving ability in people with Parkinson’s disease. J Neurol Neurosurg Psychiatry 1998; 64: 325–330. 9527142 10.1136/jnnp.64.3.325 PMC2170019

[4] Meindorfner C Körner Y Möller JC , et al. Driving in Parkinson’s disease: mobility, accidents, and sudden onset of sleep at the wheel. Mov Disord 2005; 20: 832–842. 15726539 10.1002/mds.20412

[5] Hirschtick JL Homan SM Rauscher G , et al. Persistent and aggressive interactions with the police: potential mental health implications. Epidemiol Psychiatr Sci 2019; 29: e19. 10.1017/S2045796019000015 PMC8061162 30714560

[6] Holmes JD Alvarez L Johnson AM , et al. Driving with Parkinson’s disease: exploring lived experience. Parkinsons Dis 2019; 2019: 3169679. 30854186 10.1155/2019/3169679 PMC6378012

[7] Rizzo M Uc EY Dawson J , et al. Driving difficulties in Parkinson's disease. Mov Disord 2010; 25: S136–S140. 10.1002/mds.22791 PMC3108508 20187237

[8] Richard IH Schiffer RB Kurlan R . Anxiety and Parkinson’s disease. J Neuropsychiatry Clin Neurosci 1996; 8: 383–392. 9116473 10.1176/jnp.8.4.383

[9] Sun F Gao X Brown H , et al. Police officer competence in handling Alzheimer’s cases: the roles of AD knowledge, beliefs, and exposure. Dementia 2019; 18: 674–684. 28084808 10.1177/1471301216688605

[10] Aarsland D Brønnick K Larsen JP , et al. Cognitive impairment in incident, untreated Parkinson disease: the Norwegian ParkWest study. Neurology 2009; 72: 1121–1126. 19020293 10.1212/01.wnl.0000338632.00552.cb

